# Central America in Transition: From Maize to Wheat. Challenges and Opportunities

**DOI:** 10.3390/nu7095330

**Published:** 2015-08-26

**Authors:** Amado Salvador Peña, Jakob Bart Arie Crusius

**Affiliations:** Laboratory of Immunogenetics, VU University Medical Center, Department of Medical Microbiology and Infection Control, P.O. Box 7057, 1007 MB Amsterdam, The Netherlands

**Keywords:** celiac disease, prevalence, HLA-DQ, serological tests, anti-Endomysium antibodies, tissue transglutaminase antibodies, hygiene hypothesis, old-friend hypothesis, Central America

## Abstract

The Central American countries: Guatemala, El Salvador, Honduras, Nicaragua, Costa Rica, and Panama are in transition from a dietary culture based mainly on maize to a wheat-containing diet. Several other changes are occurring, such as a decrease of parasitic and infectious diseases. The environmental changes permit a prediction of an increase of celiac disease and other autoimmune diseases such as type I diabetes and thyroid disease in these genetically heterogeneous countries. At present, celiac disease and gluten-related disorders are considered to be of no relevance at the level of public health in these nations. This review documents the presence of celiac disease in Central America. It draws attention to some of the challenges in planning systematic studies in the region since up until recently celiac disease was unknown. The aim of this review is to disseminate knowledge obtained with preliminary data, to stimulate clinical and basic scientists to study these diseases in Central America and to alert authorities responsible for the planning of education and health, to find possibilities to avoid a rise in these disorders before the epidemics start, as has occurred in the Mediterranean countries.

## 1. Introduction

Central America is the geographic region in the Americas between the subcontinents North and South America, to the north bordered by Mexico, to the southeast by Colombia, to the east by the Caribbean Sea and the Pacific Ocean to the west. It consists of seven countries: Belize, Guatemala, El Salvador, Honduras, Nicaragua, Costa Rica, and Panama. In this review, Belize has not been taken into consideration.

In Central America celiac disease and gluten-related disorders are not well known. Until recently only few confirmed celiac disease cases have been described. The majority of patients are adults diagnosed in national public health institutions. Some patients have been diagnosed in private clinics in Central American countries, Mexico or in the U.S.A. The tests used for diagnosis have been specific serological markers and intestinal biopsy through upper gastrointestinal endoscopy. According to recent available literature in only a small series of patients determination of the human leukocyte antigen (HLA)-based genetic susceptibility to celiac disease took place in El Salvador [1] and Costa Rica [2].

The staple food of Central American countries, since the pre-Columbian era consisted of maize, “plátanos maduros” (plantain) and cassava (called “yuca” in this region) together with beans and rice. Therefore, this diet was intrinsically gluten-free without possible gluten cross-contamination. Probably the commercial influence on consumer patterns in these countries, gluten-containing food is increasingly used. Environmental changes in the consumption culture are bound to have an effect on the composition of the intestinal flora. This tendency has been developing for decades, particularly after 1980. Gluten-containing products provided by welfare services or humanitarian organizations are now reaching rural areas.

At present tentative generalizations about the prevalence of celiac disease in these countries are risky, taking into account the high number of variables acting in Central America. Properly designed epidemiologic and genetic studies are necessary. The prevalence of celiac disease and gluten-related disorders may vary among these countries, even within one country the prevalence may vary by region.

## 2. Genetic and Culture Heterogeneity Are Present in the Six Central American Countries

According to a study published in Nature [3] of 52 Native American and 17 Siberian groups genotyped at 364,470 single nucleotide polymorphisms, the Native Americans descend from at least three streams of Asian gene flow. The majority descends from a single ancestral population, the “First Americans”. This initial population was followed by a southward expansion along the west coast, with subsequent divisions but with little change in the gene flow after the divergence. The Chibchas known as Muisca people are according to these authors an important exception on both sides of the Panama isthmus, since they possess ancestors from both North and South America. Much later, in the 15th and 16th century the European colonizers introduce a very diverse genetic inheritance, product of the complex history of their continent of origin. Large groups of slaves imported from Africa came primarily to the Atlantic coast of Central America, although there is evidence that some also migrated to the pacific side such as El Salvador. This history emphasizes the genetic diversity without considering the migration of the post-colonial period. For example, in the highlands of Guatemala the ethnic composition of the population is markedly different from that of the larger cities. This is due to the fact that colonial cities tended to act as administrative centers, so that European-descended people would gather there, relegating the original inhabitants to rural areas [4].

## 3. Difficulties in Diagnosis and Challenges for the Future

No epidemiological studies on celiac disease have been performed in any of the Central American countries. Some series of patients with celiac disease have been described. Their diagnosis was based on the determination in blood of specific serological tests, like the detection of IgA anti-gliadin (AGA), IgA anti-tissue transglutaminase (tTG) and/or IgA anti-Endomysium antibodies (EmA). In general the most important genetic markers of susceptibility, the HLA class-II antigens: HLA-DQ2.5 and/or HLA-DQ8 have not been taken into account. The histological findings of the duodenal biopsy specimens, based on the presence of villous atrophy and more recent on increased intraepithelial lymphocytes without villous atrophy and the response to a gluten-free diet have been taken into account to confirm the diagnosis. From the clinical point of view the inability to detect Marsh I celiac patients by specific serological tests among populations with low gluten consumption such as the rural populations of these countries is difficult. Although tropical sprue is often referred to as endemic in Central America there are no publications that suggest that it may be present. In the literature most of the patients with tropical sprue have been reported in Porto Rico, the Dominican Republic and other islands in the Caribbean [5].

In spite of the need of upper gastrointestinal endoscopy, limited availability, technical failures, lack of orientation and/or the sampling of insufficient biopsies, the “gold standard” for the diagnosis of celiac disease continues to be the small intestinal biopsy [3] in these countries. Also few pathologists appear to be interested in the proper assessment of the specimens. During endoscopy, multiple biopsies in the duodenal bulb and at least 4 in the distal duodenum should be taken. In a multicenter study carried out in children, it was confirmed that in 2.4% of 665 patients, the lesions were virtually limited to the duodenal bulb. The majority of the studies published so far do not comply with the protocol suggested by Bonamico *et al.* [6]. No data is available in Central America.

## 4. Results

Preliminary studies of celiac disease in Central America:

### 4.1. Guatemala

In Guatemala, a master thesis written by a nutritionist [7] determined the number of patients suffering from celiac disease in private clinics in gastroenterology of the capital city of Guatemala in order to develop a manual of nutritional guidance to the Guatemalan celiac patient. The results showed one case of celiac disease for every 1000 adult patients. The most frequent age of patients diagnosed was from 31 to 50 years of age, 59% were female. The “Asociación de Celíacos de Guatemala (“Celiac Patients’ Association of Guatemala”) was founded in Guatemala city in 2013 by Dr. E. Ligorría, who is deeply involved in the study of celiac disease and the treatment of local celiac patients. No data has been published yet. It is to be hoped that he will be able to organize systematic studies not only among the mestizos (people of mixed Native and European heritage) population but also among the indigenous Mayan descent population of Petén, the northernmost department of Guatemala. This population would be important to study the distribution of HLA antigens and the effects of the ongoing dietary transition from maize to gluten-containing products.

### 4.2. El Salvador

The first study using the modified Marsh classification and the full HLA-DQ typing in El Salvador has been recently published [1]. Based on serological tests, histological features of duodenal biopsy specimens and the response to a gluten-free diet, 32 individuals (23 females and 9 males) were diagnosed with celiac disease. The age at diagnosis ranges from 19 to 77 years. All patients are urban residents. Upon revision of the biopsy specimens and classification of the histological features, 28 showed the histological features that are compatible with celiac disease [6]. Twenty-three have celiac disease risk genotype: 14 HLA-DQ8 (DQA1*03/DQB1*0302; 12F and 2M), 7 HLA-DQ2.5 (DQA1*05/DQB1*02; 3F and 4M), 2 HLA-DQ2.5 and DQ8 (1F, 1M), and 9 cases (7F, 2M) who had neither DQ2.5 nor DQ8. All nine non-DQ2.5/non-DQ8 cases reported an improvement of symptoms with a gluten-free diet; two had been diagnosed abroad. Seven out of nine non-DQ2.5/non-DQ8 celiac disease patients were heterozygous carriers of allele DQA1*05 only and one had HLA-DQ2.2 (DQA1*0201/DQB1*02). One patient did not possess an HLA-DQ genotype associated with celiac disease. Another patient had also dermatitis herpetiformis.

Ethnic admixture is characteristic of El Salvador. In the ethnic categories identified in colonial times, the predominance of mestizos is important in El Salvador. Several factors influenced this outcome: (a) in El Salvador’s current territory there was no place where indigenous peoples could find refuge, so that they and the Spaniards had to coexist in the same space; (b) the decrease in the indigenous population due to diseases and massacres; (c) population break up due to its exploitation for the cultivation of indigo in the 18th and 19th centuries. In El Salvador the “Asociación de Celíacos y Sensibles al Gluten de El Salvador” (ACELYSES) (“Celiac and Gluten Sensitive Association in El Salvador”) is playing a predominant role in dissemination of knowledge. The primary mission is the dissemination of information on gluten-related disorders as well as promoting education and awareness among celiac disease and gluten-sensitive people, their families, public and private health institutions and other organizations which have an impact on the quality of life of celiac and gluten-sensitive people in El Salvador.

### 4.3. Honduras

Only isolated cases of celiac disease have been reported in Honduras. There is no celiac or gluten sensitive patient association in this country yet but the few celiacs are stimulating knowledge among the general practitioners, specialists in gastroenterology and internal medicine. They collaborate with other Celiac Associations in bordering countries.

### 4.4. Nicaragua

In Nicaragua, few cases are known. Possibly, this is due to the lack of knowledge of the disease. In the department of Managua (the capital of the country), the Faculty of Medical Sciences of the National Autonomous University of Nicaragua (UNAN-Managua) has conducted a study among students and faculty members. The knowledge that the respondents had about celiac disease was minimal. The teaching faculty had a higher percentage of those who knew about the disease and its clinical presentation. More surprisingly, 55.2% of faculty members involved in this study did not know about celiac disease [8].

### 4.5. Costa Rica

Preliminary results of 258 patients (108 male and 150 female) with celiac disease in Costa Rica with lymphocytic duodenitis and villous atrophy have been published. Mean age was 48.3 years, ranging between 16 and 90 years. Thirty-six patients were typed for the HLA-DQ2.5 and HLA-DQ8 alleles; 11 cases were positive for HLA-DQ2.5, 7 for HLA-DQ8, and 3 for both HLA-DQ2.5 and HLA-DQ8. Interestingly 15 of the 36 patients turned out to be negative, and had lymphocytic duodenitis. Further follow-up of these patients is needed since the differential diagnosis for lymphocytic duodenitis is extensive and several known causes have not yet been excluded [2].

Allele group and haplotype frequencies of HLA genes in the Costa Rica Central Valley Population, the major population of Costa Rica, have recently been determined by means of molecular typing in a sample of 130 unrelated blood donors [9]. According to these investigators the frequencies observed are consistent with a profile of a dynamic and diverse population, with a hybrid ethnic origin, predominantly Caucasian-Amerindian. The results show that the people from Costa Rica genetically are close to the Mestizo urban population from Venezuela and from Guadalajara in Mexico [9].

In Costa Rica, the “Asociación Pro-Personas Celíacas” (APPCEL) (“Pro Celiac Persons’ Association”) founded in 2004, is actively engaged offering psychological support as well as useful information by means of an e-newsletter and has been involved in changing the country’s laws so that celiac patients may have access to safe food and properly labeled products. The “Centro de Información sobre la Enfermedad Celíaca” (CIEC), “Celiac Disease Information Center” has its headquarters in San José, Costa Rica is promoting studies and provides guidance to patients with celiac disease. They coordinate with APPCEL.

### 4.6. Panamá

The “Fundación Celíacos de Panamá” (FUCEPA) (“Panama Celiac Patients’ Foundation”) is helping celiac patients and their families by means of the dissemination of information. A good coordination exists between pediatricians and gastroenterologists interested in celiac disease. No epidemiological studies or data on the number of patients have yet been published.

## 5. Discussion

In Central America, celiac disease is not considered to be relevant at the level of public health. However, understanding the epidemiology of this disease and gluten related disorders is crucial for hypothesizing about causes and quantifying the burden of disease [10]. It is well known that patients with celiac disease have a greater burden of disease than the general population because of the increase risk of osteoporosis, autoimmune diseases and malignancies. As far as we know there is only one study of epidemiology ongoing in Central America. This is in El Salvador, it covers the adult population.

The statement made 10 years ago by Green *et al.* is still valid today: “There is a need for screening studies of patients with conditions associated with celiac disease to determine whether the large numbers of people with undiagnosed celiac disease currently are seeking health care” [11]. Currently, there is a need to quantify the increase in wheat allergy, as part of the increase in allergic conditions. Also it is necessary to quantify the relevance of other gluten related disorders for the awakening of the officers of national health systems to assess the total burden of these diseases and to be prepared for the application of adequate funds.

In a recent review on the prevalence of celiac disease in Latin America [12], 72 studies were included. No publication included Central American countries. According to this review, the estimated prevalence of celiac disease in Latin Americans excluding Central America ranged between 0.46% and 0.64%. The prevalence in first-degree relatives of probands with celiac disease was 5.5%. The coexistence of celiac disease and type 1 diabetes mellitus varied from 4.6% to 8.7%, depending on the methods used for diagnosis (*i.e.*, autoantibodies and/or small intestinal biopsies). In the northern border of Central America the information published from Mexico is scarce. It seems however that in the adult Mexican Mestizo population the presence of celiac disease is relatively high [13]. A recent update [14] using the weighted prevalence for double-positive serology IgA tTG and IgA EmA the prevalence was 0.59% (95% confidence interval (CI), 0.27–1.29). A high prevalence of 5.9% biopsy-proven celiac disease was found in Mexican Mestizo patients with type 1 diabetes mellitus [15].

We wish to underscore the recommendation expressed by Gonzales Burchard *et al.*: “Precisely because of this complexity, *Latinos* present a unique opportunity to disentangle the clinical, social, environmental, and genetic underpinnings of population differences in health outcomes” [16]. 

Other observations made in Russia and Finland are relevant to understand the value of studying regions with different genetic and environmental heterogeneity. It has been found that two adjacent populations which are equally exposed to grain products and share the same ancestry but live in different socioeconomic environment such as, Russian Karelia and Finland, exhibit a different prevalence of celiac disease. The prevalence of transglutaminase antibodies and celiac disease is lower in Russian Karelia than in Finland. To explain the differences in prevalence, the authors of the study have suggested that there exists a protective environment characterized by inferior prosperity and standard of hygiene in Karelia [17]. They emphasized that: “*The lower prevalence of celiac disease in Russian Karelia seems not to be due to differences in genetic predisposition or to the consumption of grain products, but may be associated with a protective environment characterized by poorer living conditions and standard of hygiene*”.

Central America is another region in the world where the “hygiene hypothesis” [18] may apply. The improvement in the infection rates and the deworming will be responsible for an increase in celiac disease, allergy and other autoimmune diseases similar to the differences observed in Russian Karelia and in Finland. In Central America one can imagine that one of the mechanisms to explain the raise of celiac disease and other autoimmune diseases is the “old friend hypothesis” whereby the eradication of the helminthes, common in these populations, alter the numbers of T regulatory cells complementing the shift from T helper 1 (Th1) to T helper 2 (Th2) response [19,20]. In order to strengthen the awareness of celiac disease in Central America it is necessary to raise funds following the initiative of the World Gastroenterology Organization and the Asian Pacific Association of Gastroenterology in 2014 to address key issues in emergence of celiac disease in Asia [21]. Antibiotic control and more attention to the original rich staple food of Central America are two important points that may be addressed.

## 6. Conclusions

This review has shown the preliminary data that exist in Central America. It has made clear the heterogeneity of the populations involved and the lack of knowledge that still exists in this region. It is important to know in the planning of genetic studies that methods for genotyping HLA-DQ may not be suitable to detect the genetic variants of people with different ancestry. Therefore, the results should be validated with local populations before drawing conclusions. The collaboration of clinicians, immunologists, geneticists, pathologists, nutritionists and epidemiologists is essential to integrate the necessary knowledge on the diagnoses of these diseases in Central America and in helping to implement measures to avoid the emergence of new cases. As Greco *et al.* have estimated: “*In the near future, the burden of celiac disease will increase tremendously. Few Mediterranean countries are able to face this expanding epidemic*” [22]. Only a multidisciplinary approach may prevent a similar situation in Central America.
